# Screened-Room Measurements on the NIST Spherical-Dipole Standard Radiator

**DOI:** 10.6028/jres.099.066

**Published:** 1994

**Authors:** G. Koepke, J. Randa

**Affiliations:** National Institute of Standards and Technology, Boulder, CO 80303

**Keywords:** MIL-STD-462, radiated emissions, screened room, spherical dipole, standard radiator

## Abstract

We report the results of a study of measurements of radiated emissions from the NIST sphcrical-dipole standard radiator in several screened rooms. The study serves as a demonstration of possible applications of the standard radiator as well as an investigation of radiated-emissions measurements in screened rooms. The screened-room measurements were performed in accordance with MIL-STD)-462 (1967). Large differences occurred in the field intensity measured at different laboratories and even on different days at the same laboratory. There was a systematic difference at low frequencies between the screened-room results and results obtained in a transverse electromagnetic (TEM) cell, open-area test site (OATS), and anechoic chamber. We also present the results of OATS tests confirming the temporal stability of the standard radiator and measuring the loading effect of a ground plane as a function of distance from the sphere.

## 1. Introduction

The National Institute of Standards and Technology has recently developed a spherical-dipole standard radiator for use in electromagnetic interference and compatibility (EMI/EMC) applications. The design, construction, and operation of the device are described in Refs. [1,2], which also present results of tests in various NIST facilities – the open area test site (OATS), anechoic chamber (AC), transverse electromagnetic (TEM) cell, and mode-stirred chamber. The spherical radiator is a well controlled, well characterized source of electromagnetic radiation for the frequency range between about 5 MHz and over 1 GHz. As such, it can be used to test the ability of a laboratory to measure radiated electromagnetic emissions. That, in fact, was one of the principal motivations for the development of the standard radiator. It can also be used to compare different test methods, to test the validity of new measurement techniques, in round-robin intercomparisons among many laboratories, or as a check standard to confirm day-to-day repeatability at a single laboratory. The study reported below demonstrates several of these possible uses of the standard radiator. A preliminary account of the results is contained in Ref. [3]. The application of initial interest was in the competence testing of laboratories seeking accreditation for radiated emissions testing, but in the course of the study the standard radiator was also used as a known source to assess the basic test method, as a check standard, and as the test artifact in a multi-laboratory intercomparison.

In this paper we consider radiated-emissions measurements performed on a spherical-dipole standard radiator in three different screened rooms. The original goal was to develop procedures for using the NIST spherical-dipole standard radiator in the laboratory accreditation process, particularly in the National Voluntary Laboratory Accreditation Program (NVLAP) for accrediting laboratories performing MIL-STD-462 acceptance testing. To this end, we sought and received the cooperation of three EMC test laboratories to perform MIL-STD-462 RE02 tests on the spherical radiator. The intent was to establish a baseline of performance for the radiator, against which measurements at other laboratories could be compared in order to assess their ability to perform MIL-STD-462 tests. Tests at NIST had already characterized the performance of the spherical-dipole standard radiators in test facilities simulating quasi-free-space environments (OATS, AC, TEM) and in the mode-stirred chamber, but the radiators had not been tested in screened rooms, which are the common environment for MIL-STD-462 tests.

Measurements in screened rooms have a (well-deserved) tarnished reputation. We will not examine in detail the causes of the problems of screened-room measurements, but a few comments are useful as background. Our remarks will address the case of radiated emissions, but analogous effects occur for radiated susceptibility. There are many sources of potential errors in EMI measurements inside screened rooms. Perhaps the most obvious effect is that a screened room is a conducting cavity, and thus it exhibits cavity resonances and standing waves. Consequently, the field distribution within the room generally is nonuniform, and the field intensity measured depends on the locations of the equipment under test (EUT) and the measuring antenna, as well as on the electrical size of the room. Another potential source of error is that the behavior of the receiving antenna is affected by the proximity of the conducting walls. The interactions between the antenna and its numerous images change the antenna factor, and consequently the antenna response in a given electric field depends on the antenna’s location, the size of the room, and the type of antenna. A similar effect can occur for the EUT. If we think of the EUT as a transmitting antenna, its input impedance will be changed by the interaction with its images, thereby changing the ratio of terminal voltage to input current. Thus the radiated power can depend on the size of the room, the EUT’s position in the room, and details of the EUT itself. (The loading effect on the standard radiator will be addressed below.) Finally, most screened-room measurements are done at low enough frequencies that the EUT and the receiving antenna are in each other’s near fields.

The potential problems with screened-room measurements have been widely appreciated for some time [4–7]. Nevertheless, screened rooms are widely used in EMI/EMC. Their appeal is partly economic, partly inertial, and partly due to the fact that competing techniques are not without problems of their own. Open-area test sites admit background noise; anechoic chambers are expensive and become echoic at low frequencies; TEM cells have high-frequency cutoffs and size constraints; etc. Screened rooms are particularly prevalent in MIL-STD-462 testing [8], where their use is nearly universal. An extensive revision of MIL-STD-461/462 has recently been released, which contains (among other things) changes intended to improve screened room test methods [9]. The revised standard is labeled MIL-STD-462D. The tests described in this report were performed according to the old standard, MIL-STD-462 (1967), since the contents of the new one were not known at the time of the tests. We will discuss this below.

Over the course of a year, radiated-emissions tests were performed at the three participating EMC labs. All three screened rooms had absorber loading to some degree, and all were large enough to meet MIL-STD-462 (1967) specifications. We do not detail the actual sizes and specific configurations of the individual rooms. That information would be needed for diagnosing the cause of inter-laboratory differences, for example, but for this study we are just interested in the fact that they did conform to the (old) MIL-STD requirements. (There was not enough absorber in any of the screened rooms to meet the requirements of MIL-STD-462D [9].) Each set of measurements was performed twice at each laboratory, with the setup disassembled between the two measurements, in order to evaluate the repeatability of the tests. We were thus able to address three major issues: day-to-day variations at a given laboratory, differences between results obtained at different laboratories, and differences between the screened-room results and results obtained at NIST in simulated free-space environments. The results caused us to reconsider the appropriateness of using the standard radiators in the accreditation process for MIL-STD-462 measurements (under the old standard). The differences in all three areas – day-to-day variations, interlaboratory variations, and screened-room to free-space differences–were sufficiently large that the basic validity of the old RE02 test procedures in a screened room must be questioned. This point is addressed in the final section below. In the next section we review the general design of the spherical dipole radiator and present results of measurements at NIST facilities. In Section 3 we outline the procedures followed in the screened-room (RE02) tests on the standard radiator and present the results of those tests. Section 4 contains a discussion of the results and conclusions.

## 2. The Spherical-Dipole Standard Radiator

**Fig. 1:**
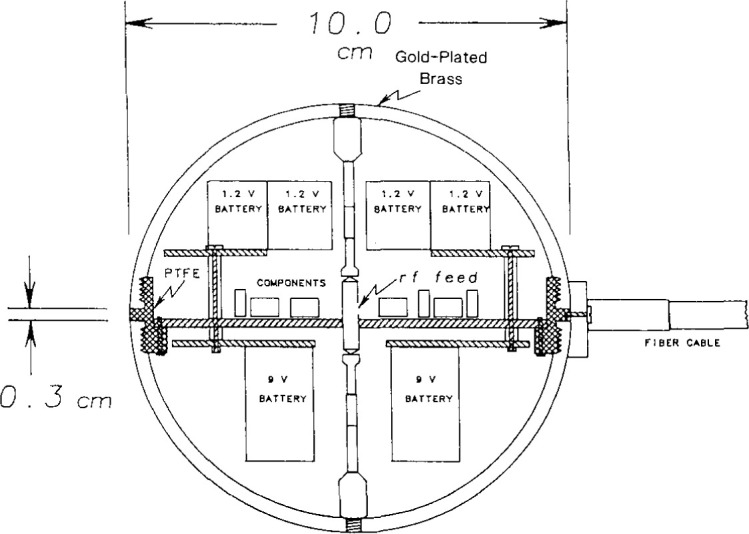
Mechanical drawing of the spherical dipole radiator.

The spherical-dipole radiator is described in detail in Refs. [1,2]. For present purposes, it is sufficient to recall a few of its principal features. The radiating element is a spherical, gold-plated dipole of 10 cm diameter, the basic configuration of which is indicated in Fig. 1. The driving voltage is applied at the gap between the center posts, and the current flows up the top post to the inside top of the sphere and down the bottom post to the inside bottom of the sphere. From the poles of the inside of the sphere, the current flows on the inner surface of the sphere out to the equatorial gap, where it feeds the outer surface of the sphere. Thus, provided that the current propagates from the rf feed uniformly to all points on the equator, we have a center-fed spherical dipole, uniformly excited around its equator. The voltage at the gap of the center post is monitored continuously by a diode detector circuit, and this reading is relayed back to the control unit via optical fiber. This feature enables the operator to verify that the impressed voltage is the same from one test to another, and it also confirms that the unit is operating properly throughout a set of measurements.

The excitation waveform is fed to the sphere by an optical fiber. Inside the sphere the optical signal is converted to an electrical signal, amplified, and fed to the gap in the center post. In the tests described in this report, a single-frequency cw signal was always used. In principle, virtually any waveform could be used to drive the spherical dipole, though the radiated waveform would include the shaping effect of the sphere’s frequency-dependent radiation characteristics. The pulse characteristics of the spherical dipole radiator have not yet been examined.

**Fig. 2:**
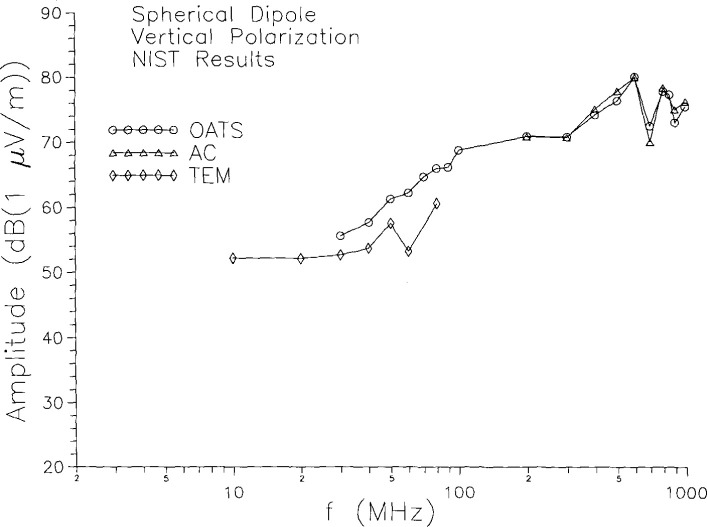
Calculated field at 1 m distance using transfer function measured at NIST facilities.

Detailed tests of the angular pattern and the intensity of the radiated field were reported in [1,2], and we do not reproduce them all here. One aspect of those tests which is relevant to the present study is the determination of the radiated field intensity. Although the voltage across the gap in the center post is continuously monitored, it does not enable us to directly calculate the radiated field, since the relationship between the voltage at the post gap and the voltage at the equatorial gap in the spherical shell cannot be easily calculated. Therefore the transfer function between the post gap voltage and the radiated field was determined empirically. This was done by measurements on the NIST OATS and in the AC. For a (post) gap voltage of 1 V, the maximum field intensity was measured at some convenient distance from the sphere. The known radiation characteristics of a spherical dipole were then used to calculate the voltage at the equatorial gap. In the AC the free-space formula for the radiation pattern was used, whereas on the OATS the effect of the ground plane was taken into account. Based on those tests, a transfer function which relates the indicated post gap voltage to the radiated field intensity (in free space) was obtained. Using this measured transfer function, we can then compute the field intensity for a given indication of the gap voltage and a given position. Figure 2 plots the field as a function of frequency for a position in the equatorial plane, 1 m from the radiator. Besides the OATS and AC results, Fig. 2 also contains the results of analogous measurements in a TEM cell [1]. The results from the OATS and AC agree very well in their region of overlap (200 MHz to 1000 MHz). The TEM cell results fall below those of the OATS by about 2 dB to 4 dB (except at one anomalous point). This difference may be due to the loading effect of the TEM cell walls on the sphere, since the radiator was about 30 cm from the walls in the TEM cell measurements. The possible effects of loading are addressed below.

**Fig. 3:**
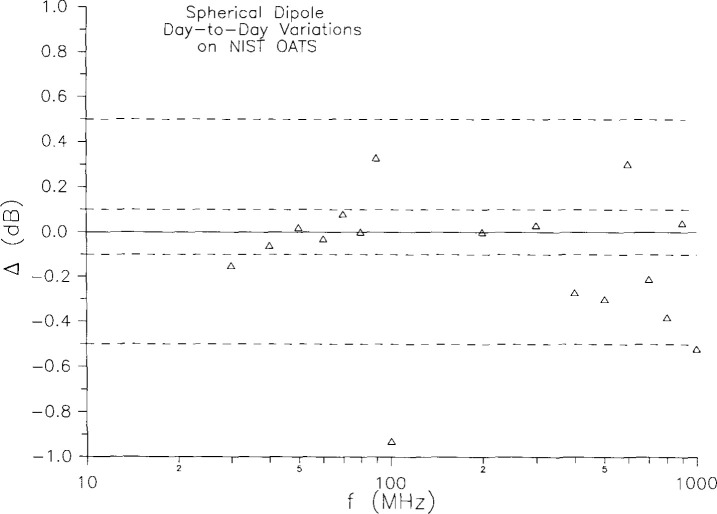
Day-to-day variations in radiated emissions measurements on standard radiator on NIST OATS.

For virtually all standard-radiator applications, and in particular for the screened-room study reported in this paper, the radiator’s repeatability is a crucial issue. We must be confident that the spherical dipole is constant if we are to use it to compare measurement results taken at different times. There is some evidence for the spherical dipole’s repeatability in the agreement of AC and OATS results in Fig. 2. We have now performed a systematic test which confirms this point. Measurements of the field radiated by the spherical dipole were made on the NIST OATS on two different days, with the measurement apparatus disassembled and reassembled between the two sets of measurements. The gap voltage was maintained at (1.00 ±0.01) V; the dipole axis was horizontal; it was about 2 m above the ground plane; and the distance from the sphere to the receiving antenna was between 7 m and 8 m in each case. The receiving antenna (a calibrated, tuned dipole) was positioned 2 m above the ground screen, provided that a usable signal was obtained at that height. For frequencies at which the 2 m height corresponded to a null of the pattern arising from interference between direct and reflected waves, the receiving height was increased until a usable signal was obtained. The measured field was converted to a field intensity at 1 m, called *E*_ref_. This was done in the manner described above, except that the full expression was used for the field from the image, rather than just the ray approximation of Ref. [1]. The difference *Δ* between the values measured for *E*_ref_ on the two different days is plotted as a function of frequency in Fig. 3. The repeatability is very good, better than 0.55 dB at all but one measurement frequency and better than 0.1 dB at almost half the frequencies. Even at the one “bad” point (100 MHz), the difference is 0.93 dB. Such variations are consistent with what we expect from the measurement method itself; our OATS measurements have a statistical uncertainty characterized by a standard deviation of about 0.5 dB. Thus the variations in the field radiated by the spherical dipole (for a constant gap voltage) are less than or about equal to 0.5 dB, and could be significantly less.

**Fig. 4:**
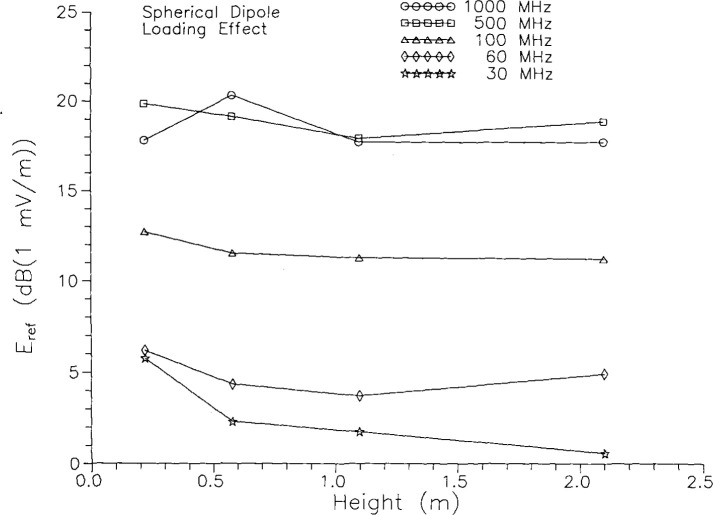
Field radiated by spherical dipole as a function of height above ground plane.

The final aspect of the standard radiator’s performance which is important to the screened-room study is the effect of loading, the sensitivity of the radiator to nearby conducting surfaces. Measurements at and above 100 MHz in the mode-stirred chamber [2] did not show evidence of a loading effect on the spherical dipole for a dipole-to-wall separation of 1 m, at least within the accuracy of the measurements, and the TEM cell results in Fig. 2 suggest that the effect is of order a few decibels for a distance of 30 cm. We have now also performed a series of measurements on the OATS for several different heights of the sphere above the ground screen. At five frequencies, from 30 MHz to 1000 MHz, the radiated field intensity was measured for four heights, *h*_t_, ranging from 0.22 m to 2.1 m. The gap voltage was 1.00 V in all the measurements, and the measured field was converted to *E*_ref_, the field at 1 m. Results are shown in Fig. 4. The uncertainty in the measurements is about 1 dB. At the lowest frequency, 30 MHz, there is a definite increase in the radiated field for *h*_t_ < 0.5 m. At other frequencies there is no clear evidence for an effect of loading, although something may be happening around *h*_t_ = 0.5 m at 1000 MHz and for *h*_t_<0.5 m at 60 MHz. For *h*_t_ ≥ 1 m, the data do not indicate a loading effect at any frequency tested, although we cannot rule out an effect of order 1 dB-2 dB. In the screened-room measurements discussed below, the sphere was never closer to a wall than 1 m.

## 3. Screened-Room Measurements

### 3.1 Procedures

The three participating laboratories will not be identified in discussion of the results, and only aggregate data will be shown. At the time of the tests, one of the laboratories was NVLAP certified for MIL-STD-462 acceptance testing, and the other two were working toward certification. Tests were performed over two days at each laboratory. The same spherical-dipole unit was used in all the tests. The intent was that the NIST dipole radiator would be treated as if it were a piece of electronic equipment submitted to the laboratory for RE02 acceptance testing. The dipole was to be treated as a piece of mobile equipment placed on a foam support out in the room. Tests were also performed on a small, battery-operated monopole radiator [3], which was tested on the bench top/ground plane. However, subsequent tests revealed possible problems with the monopole’s repeatability, and so we will not present results of the monopole measurements in the screened rooms. In all the tests the radiator was oriented so that its axis was vertical. For low frequencies (below 20 MHz or 30 MHz, depending on the laljoratory), the receiving antenna was a small monopole, and only the vertical component of the radiated field was measured. From 20 MHz or 30 MHz to 200 MHz, a biconical antenna was used, and vertical and horizontal components were measured separately. Above 200 MHz, all three laboratories used conical log-spiral antennas, sensitive to one circular polarization.

At 20 MHz, 30 MHz, and 200 MHz, one or more laboratories changed the receiving antenna used. At these frequencies, measurements were taken on radiated signals at frequencies at the top of the lower band and at the bottom of the upper band (e.g., 19.95 MHz and 20.05 MHz) at the laboratory changing antennas at that frequency. If a laboratory did not change antennas at that frequency, then just one measurement was taken (e.g., 20.00 MHz). For the computations in which measurements from different laboratories were paired or compared, the 20.00 MHz measurement was paired or compared with both 19.95 MHz and 20.05 MHz results.

The spherical-dipole radiator was fed with a single frequency at a time, with the frequencies chosen to correspond to those at which the radiator had been tested in NIST facilities. The engineer or technician performing the test was told the frequency, and he or she swept the receiver through a small range of frequencies around the frequency being radiated. The test frequencies for the dipole ranged from 5 MHz to 1000 MHz. The gap voltage of the dipole was maintained at the same value (1.00 V) for all the tests. Each measurement at each frequency was done twice at each laboratory, typically on successive days, but in some cases in the morning and afternoon of the same day. Between the two measurements the setup was always taken down, connections broken, etc., to insure that the two measurements were as independent as was practical. In at least one case, the positioning of antennas was intentionally altered somewhat, to simulate the variations in placement which could occur in different tests. Thus we generally have two independent measurements at each frequency (and each polarization, where prescribed by the MIL-STD) at each of the three participating laboratories. Insofar as was possible, NIST personnel attempted not to influence the actual conduct of the tests. Some interaction did occur, of course, but we do not believe that there were any substantive effects on our general results.

### 3.2 Results

**Fig. 5:**
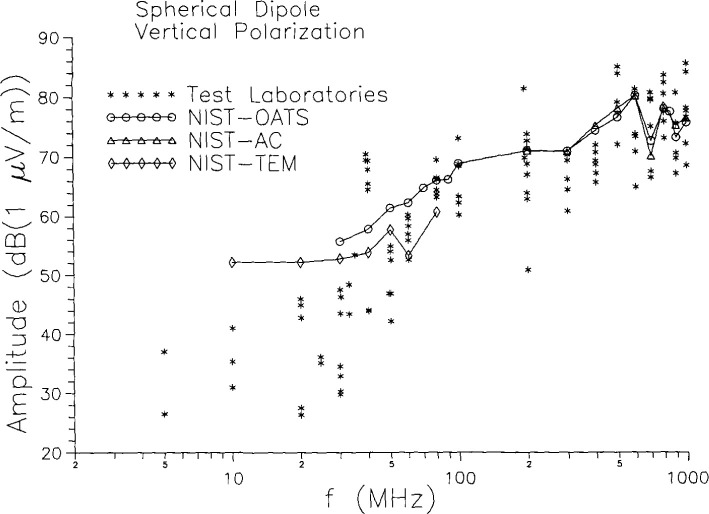
Measurement results on spherical dipole standard radiator with constant gap voltage.

The collected results of the measurements on the spherical-dipole radiator at all three laboratories, for a vertically polarized receiving antenna, are shown in Fig. 5. Low-frequency (<30 MHz) results from one of the laboratories were not available because of an error in assembling an antenna. The error was discovered during the tests, but too late to repeat the measurements. Also shown in Fig. 5 are the results obtained in the NIST facilities which simulate (to differing degrees) a free-space environment. The NIST results are connected by solid lines. The single most striking feature of Fig. 5 is the large spread in the screened-room measurement results. The differences between maximum and minimum values for the radiated field strengths at different labs are as large as 25 dB to 30 dB at some frequencies, and they are of order 10 dB even at the “good” frequencies. Figure 5 also indicates that there are often large differences between the shielded room results and the results from TEM cell, OATS, and AC. Differences between the two measurements at the same frequency at the same laboratory cannot be seen in Fig. 5, but these also can be sizable.

**Fig. 6:**
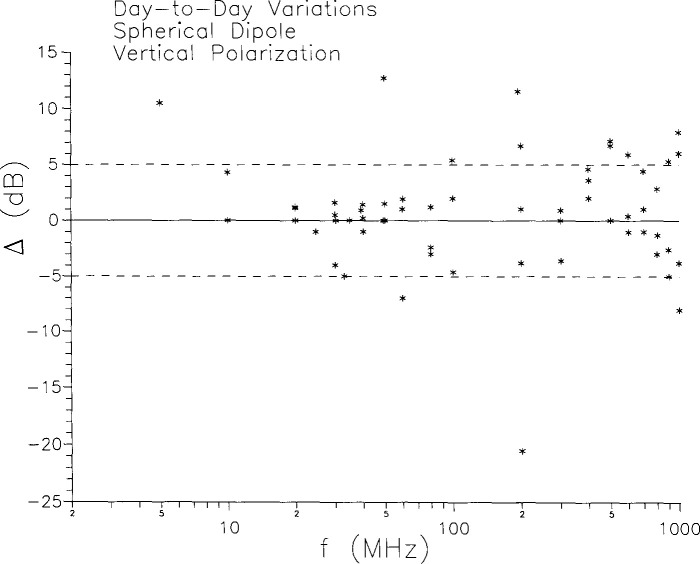
Day-to-day variations in vertical polarization measurements at the same laboratory.

For purposes of addressing separately the three different types of variations (day-to-day, interlaboratory, screened room to free space) it is useful to present the data in different formats. To address the question of repeatability of results at a given laboratory, we simply compute the difference, in decibels, between the two independent measurements at each frequency at that laboratory. This difference, denoted Δ, is plotted in Fig. 6 for all three laboratories. The dashed lines at ± 5 dB are included to aid the eye and facilitate discussion. As can be seen, most measurements repeated to within 5 dB, but a significant number (23%) did not, and some day-to-day variations exceeded 10 dB.

**Fig. 7:**
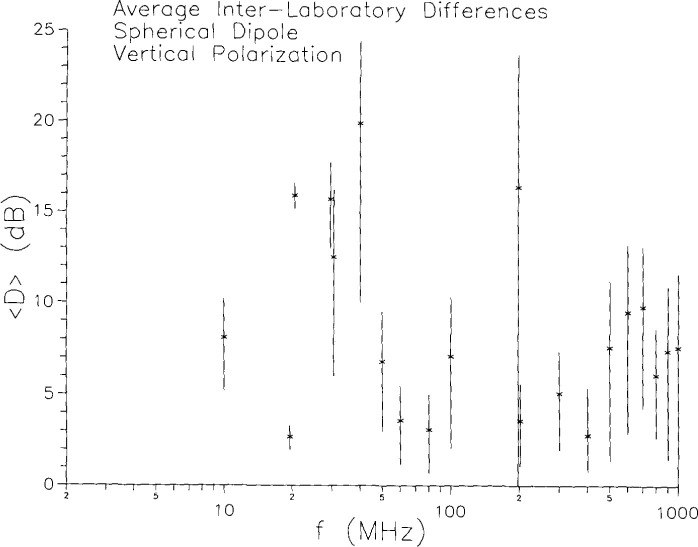
Interlaboratory differences in vertical polarization emissions measurements on the spherical dipole for constant gap voltage.

For interlaboratory variations, there are several ways in which the data might be presented. Our choice is guided by the question, “If the same measurement were made on the same device at two different laboratories, how much would the two results differ?” To answer this, we have computed at each frequency the magnitude of the difference (in decibels) between each possible pair of measurements at different laboratories. Thus, at a typical frequency, where there are two measurements at each of the three laboratories, there would be 12 different pairs of measurements at different laboratories. We use *D* to denote the difference between two measurements at different labs. Figure 7 shows the average and sample standard deviation *s*,

$$s^{2}=\frac{1}{\left(N-1\right)}{\sum_{i}\left(x_{i}-\bar{x}\right)}^{2},$$ (1)

of these differences for each measurement frequency. The statistics were done on the field strength, and the results were then converted to decibels. Results below 30 MHz are based on measurements at only two laboratories. Even above 30 MHz, the sample is not large enough for real statistical significance. Nevertheless, the results are not particularly encouraging. The *average* differences in the measurement of the same quantity at two different laboratories are over 5 dB at most frequencies and over 15 dB in some cases.

**Fig. 8:**
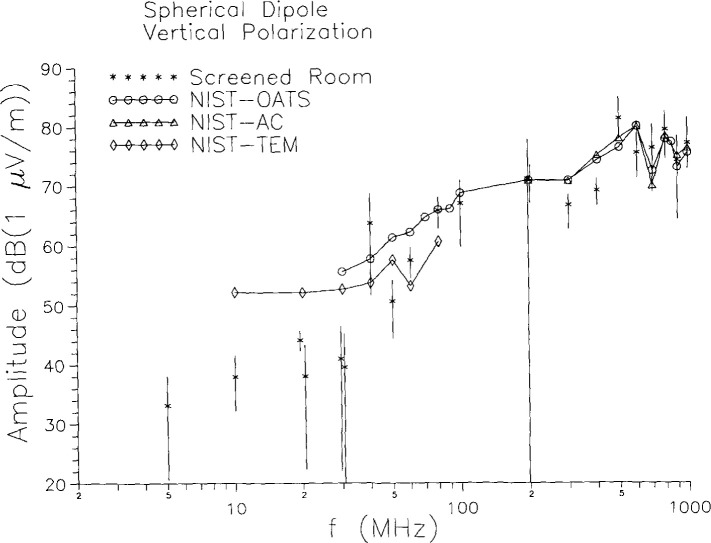
Combined screened room results compared to quasi-free-space results for vertical polarization measurements on the spherical dipole.

To consider differences between screened-room results and those obtained at quasi-free-space facilities, we average over the screened-room results obtained at the three EMC labs and compare to the TEM cell, OATS, and AC results at NIST. The results are shown in Fig. 8, where again the error bars correspond to the sample standard deviation. At high frequencies the screened-room results are in fair agreement with the quasi-free-space results, although the spread in the screened-room results is rather large. Below about 80 MHz the screened-room results tend to be systematically, significantly low. The one exception occurs at 40 MHz, which corresponds to a resonance frequency of two of the screened rooms and where the results in those two rooms are anomalously high, cf. Fig. 5. The other eye-catching feature of Fig. 8 is the large standard deviation just below the band edge at 200 MHz. The spread in the measurements at this point is so great that the results are essentially consistent with any result from 0 *V/*m (− ∞ dB) to the top of the bar shown on the graph.

**Fig. 9:**
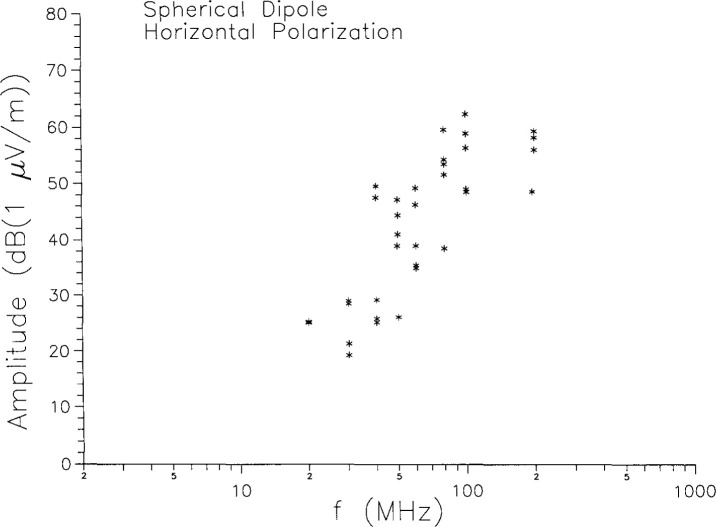
Horizontal polarization emissions measurements on the spherical dipole.

For frequencies between 20 MHz or 30 MHz and 200 MHz, emission measurements were also made with the receiving antenna horizontally polarized. The measured amplitude are shown in Fig. 9. Unlike the results for vertical polarization, there are no results shown from NIST quasi-free-space facilities. That is because the electric field from a vertical spherical dipole in free space has no component in the (*ϕ*) direction and no horizontal component at all in the equatorial plane [10]. This was checked in a few instances in the NIST facilities, and no significant field was detected. Thus the horizontally polarized fields of Fig. 9 are an artifact of performing the measurements in a screened room.

**Fig. 10:**
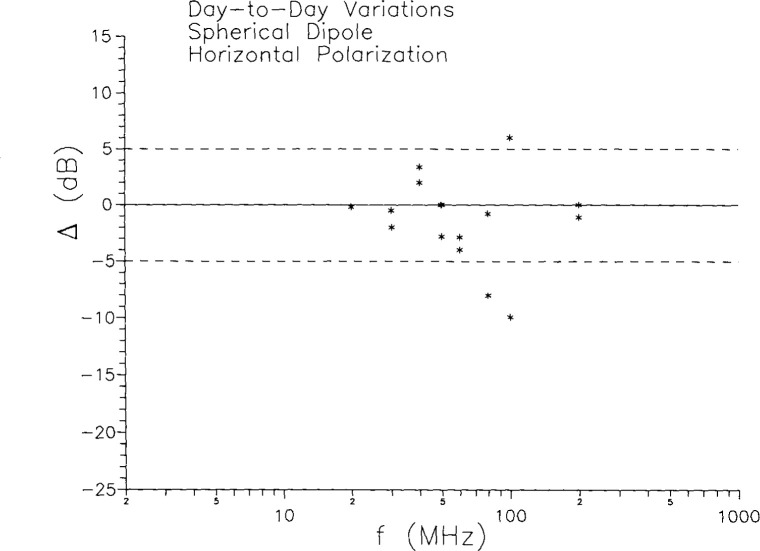
Day-to-day variations in horizontal polarization measurements at the same laboratory.

**Fig. 11:**
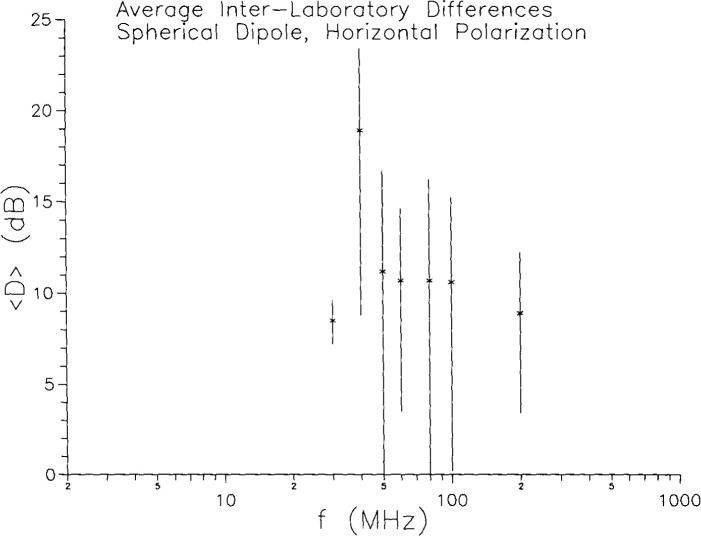
Interlaboratory differences in horizontal polarization measurements on spherical dipoie for constant gap voltage.

The day-to-day and interlaboratory variations in the results for horizontal polarization can be treated in the same manner as they were for vertical polarization. The results are shown in Figs. 10 and 11. The day-to-day variations are somewhat larger than the vertical case, as are the lab-to-lab differences. The *average* interlaboratory differences are all around 10 dB, except at the 40 MHz resonance, where they are considerably more. Since the horizontally polarized fields are basically room effects, it is not surprising that there is considerable variation from room to room. Note that if the source were a horizontal dipole, then it would be the vertical fields which arose from room effects. In general, it is the cross-polarized configuration which is due to room effects.

## 4. Summary and Conclusions

### 4.1 Screened-Room Measurements

The spherical-dipole standard radiator was used to assess the repeatability of screened-room measurements and to compare screened-room radiated-emission measurements to those made in other facilities. We first address the three main types of variations discussed in the introduction. The emissions tests, performed according to MIL-STD-462 (1967), lead us to the following conclusions. (1) Day-to-day variations of about 5 dB or more occur in measurements of radiated fields of the same magnitude, frequency, and polarization. Consistent repeatability of 5 dB or less may be achievable, but probably requires considerable effort and care. (2) The average difference between measurements of radiated, vertically polarized, electric fields of the same magnitude at different laboratories was over 10 dB at several frequencies. It is about 20 dB at a resonance frequency of one of the screened rooms. For horizontal polarization (with a vertically polarized source) the average difference is consistently around 10 dB, except at the resonance frequency, where it is near 20 dB. (3) At frequencies below about 60 MHz, the screened-room results are significantly lower than the quasi-free-space results, except at the resonance frequency of the screened room. For frequencies of 80 MHz and above, the average screened-room results are usually consistent with the quasi-free-space results, albeit with large standard deviations.

What is the cause of the large variations in test results? There are three obvious suspects: variability of the standard radiator, lack of competence of the test laboratories, and faulty test methodology (pathologies of RE02 screened-room measurements). It is very unlikely that the spherical-dipole radiator is that unstable. The gap voltage is monitored continually and is kept constant within 0.1 dB from test to test. Measurements on the NIST OATS indicated a repeatability for radiated-emission measurements of about 0.5 dB. At high frequencies there is some departure from axial symmetry in the radiated pattern [1,2], but this is only of order 3 dB to 5 dB, and it occurs only at 600 MHz and above. Furthermore, the pattern is very repeatable, even at frequencies where it is asymmetric, and in the screened-room tests the orientation of the sphere was usually the same at a given laboratory, due to positioning of the fibers running into the sphere. As for the test laboratories, in principle it is possible that they were careless or incompetent in their measurements, but we feel that this is unlikely. NIST personnel present at the tests were not trained or experienced specifically in RE02 measurement techniques and did not attempt a systematic, critical evaluation of laboratory procedures, but our impression was that laboratory personnel were in general competent and careful. As mentioned above, one of the three laboratories was NVLAP certified; and all three are large, reputable laboratories with considerable experience. Furthermore, no one laboratory stood out as having particularly bad results. Consequently, while an individual bad result could have been due to an error, we do not believe that the general pattern of variability was due to shortcomings of the laboratories or their staffs. The most likely cause of the variability of results and the deviation from free-space results appears to be the basic measurement method itself. Variations in size, shape, and loading of the screened room as well as in positioning of the source and receiving antenna within the room will lead to variations in the measured field. The existence of such effects has been known for some time and has been documented by past work at NIST [6,7] and elsewhere [4,5]. This study quantifies the magnitude of the effects in some practical measurements.

Besides the three central issues discussed above, two peripheral points which arose in this study warrant comment. The comments involve band edges. At one of the laboratories, the software and/or instrumentation were such that a peak occurring at a band edge could be missed. This problem was noted by the laboratory in question at the time of the tests. Another, more general, band edge problem is the fact that measurements in two adjoining bands do not match at the limiting frequency. In this study we found discrepancies as large as 10 dB to 20 dB at band edges. It would be desirable for the limiting frequency (at least) to be included in both bands and for techniques and calibrations to be checked until the results of the two bands agree at the limiting frequency.

In discussing the implications of our results, we emphasize that they *do not* apply to the new radiated emissions measurements as specified in [9]. The new standard incorporates modifications intended to improve various test procedures. In particular, it requires a large amount of absorber around the test setup, nearly transforming the screened room into a semianechoic chamber. The minimum acceptable performance specified for the absorber is rather modest, as it must be if anyone is to meet it; conventional absorbing materials do not perform very well at low frequencies. It is therefore not yet clear how much improvement the new standard will produce. It is clear, however, that the results of our present study do not apply to measurements in rooms meeting the new standard. They apply only to the old standard, but are significant nonetheless. For one thing, they provide a reference against which a similar study of the new standard could be compared. Such a comparison would measure how much the new standard has improved the test methods. Another consideration is that it will probably be some time before tests according to the old standard are phased out entirely. As long as the old test setup is being used, it is important that the people performing the tests–or accepting the test results – realize how accurate those results are or are not. Finally, screened rooms are used for measurements other than MIL-STD tests, and we expect the qualitative features of our study to be common to other screened-room radiated emissions measurements between 2 MHz and 1000 MHz unless special precautions are taken to mitigate the problems.

### 4.2 Applications of the Standard Radiator

We noted in the introduction that the spherical-dipole standard radiator has several possible applications, and this paper has impinged on a number of them. The initial purpose of this study was to develop procedures for using the NIST spherical-dipole standard radiator in the accreditation of laboratories doing MIL-STD-462 acceptance testing. The basic idea was to use the spherical dipole as a standard radiator to test whether the laboratory could get the “correct” answer in its radiated emissions measurements. It is clear that the radiator could be used in this manner, but the goal of developing a protocol for MIL-STD-462 accreditation was not achieved. For one thing, the standard changed, and our data are not representative of results which would be obtained with the new standard. Even for the old standard, the wide disparity in the results at different labs and even at the same lab on different days led us to conclude that proficiency testing with the NIST spherical-dipole standard radiator would be pointless. Any proficiency testing would have to allow a tolerance of around 15 dB to take into account “reasonable” variations in test results. Such crude testing would not require the precision, sophistication, and concomitant expense of the spherical-dipole standard radiator.

The study does provide a good example of how the standard radiator can be used to assess the validity of a test method–by comparing results to those obtained with accepted test methods and by evaluating repeatability, both day-to-day and laboratory to laboratory. Our data constitute a clear, quantitative demonstration of the shortcomings of radiated emissions measurements in screened rooms. It would now be of great interest to perform a similar study on radiated emissions measured in conformance with the new MIL-STD. Comparison of the results of the new study to those of the old would show how much improvement the changes made.

Another application which is clearly demonstrated in the paper is use of the standard radiator by a group of laboratories in a round-robin intercomparison of radiated-emission measurements. The spherical dipole is very well suited for this purpose due to its temporal stability, its known radiation pattern, the capability of monitoring the gap voltage, and the flexibility offered in the choice of radiated frequency. As shown by the measurements of day-to-day variations, the spherical dipole can also be used by an individual laboratory as a check standard, to check that their measurement system has not changed from day to day, or to refine their measurement procedures in order to improve the repeatability of their measurements. (Commercially produced monopole radiators offer a simpler, less expensive alternative, though they are not as well characterized or as flexible.) Finally, although it was not demonstrated in this paper, we note that the standard radiator could even be incorporated into a test procedure, for example, as a standard source for the calibration of antennas.
